# Assessing public support for air pollution mitigation and control policies: health, socioeconomic, and ideological predictors in an overburdened and vulnerable region of the U.S.

**DOI:** 10.1186/s12889-025-21366-7

**Published:** 2025-01-22

**Authors:** Gilda Zarate-Gonzalez, Paul Brown, Ricardo Cisneros

**Affiliations:** Department of Public Health, University of California, Merced, 5200 N Lake Road, Merced, CA 95343 USA

**Keywords:** Air pollution, Asthma, Quality-of-life, Equity, Policy, Health economics

## Abstract

**Background:**

The San Joaquin Valley (SJV) in California is one of the most polluted regions in the U.S. This study examined favorability for air pollution mitigation policies, interventions, and identified predictors amongst region’s residents.

**Methods:**

A cross-sectional online survey asked about health status and conditions, self-protective behaviors, attitudes toward air pollution recommendations, air quality information knowledge and mitigation behaviors, as well as whether their views were favorable or unfavorable towards policy and interventions. EuroQOL-5D-3L was utilized to estimate quality-of-life distribution equity and air pollution policy favorability.

**Results:**

A total of 310 adults residing in the SJV participated in the survey. The mean age was 42.90 years, with 34% having asthma. People with asthma reported the lowest health-related quality of life (HRQoL) compared to other air pollution “sensitive” groups. Concerns included the costs, charge and attributes of adopting hybrid or electric vehicles. Residents supported air pollution control and public space preservation, with socioeconomic factors and health state being significant predictors. Left-wing ideologies favor policies charging polluters, controlling emissions, and preserving public spaces, whereas right-wing views negatively predict support for electric vehicle charges and local organization interventions.

**Conclusions:**

Results may help air pollution control policymakers, public health agencies, environmental justice organizations, and the health equity research community understand the reasons for differential responses to air pollution interventions and mitigation efforts. This new knowledge could assist stakeholders in recommending sustainable and cost-effective interventions for improving air quality, elicit behavior change, and climate change adaptation in the region.

**Supplementary Information:**

The online version contains supplementary material available at 10.1186/s12889-025-21366-7.

## Introduction

Despite air quality standards and structural regulations to control emissions, the San Joaquin Valley (SJV) is one of the most polluted regions in the U.S., exceeding state and national standards for ground-level ozone (O_3_) and fine particulate matter (PM_2.5_) [6, 17, 46]. In 2022 and 2023, the American Lung Association gave every major city in the SJV a failing grade, F, in their ‘State of the Air’ report for high ozone days, as well as for 24-h and annual PM_2.5_ pollution [3, 5]. NO_2_ has achieved federal and state air quality standard attainment, but novel regional research supports that the criteria pollutant, a precursor of ground-level ozone, is associated with increased asthma incidence and adverse respiratory symptom visits to emergency departments and hospitalizations [15, 65, 73, 74].

Years of accumulating evidence support that outdoor air pollution is a significant contributor to asthma development [31, 67] and exacerbation [28]. Furthermore, the U.S. Environmental Protection Agency has deemed it a likely cause of respiratory disease [19]. There is evidence that deterioration in air quality is associated with increased polyclinic attendance for upper respiratory tract infections, acute conjunctivitis, lung disease, asthma, bronchitis, emphysema, and pneumonia [60]. A recent health economics study for the region concluded that air pollution of three criteria pollutants costs SJV residents US$ 1.16 billion in asthma and adverse respiratory outcomes-related emergency department visits and hospitalizations, productivity loss, missed school, and opportunity costs [73, 74]. Thus, communities have much more work to do to prevent air pollution-related health problems and improve adverse health outcomes in the immediate term.

### Region and air pollution adverse health outcomes

California’s Central San Joaquin Valley (SJV) is an air basin with unique meteorological conditions that, combined with its arid and semiarid geography, traps pollution emitted by sources and gusted from contiguous regions [57]. The region is situated at the midpoint of two areas with high population concentrations, the San Francisco Bay Area and Los Angeles [37], and it is home to approximately 4.3 million residents [54].

Environmental epidemiological studies conducted for SJV by regional researchers have revealed a positive association between short-term, multiday, ground-level ozone exposure and increased emergency department visits during the warm season for asthma and respiratory outcomes [17, 73, 74], especially in children aged 6–18 years (OR: 1.219) and people of color (OR: 1.159) [26]. In addition, short-term exposure to NO_2_ has been linked to visits to the emergency room due to asthma exacerbations during both the cold and warm seasons [15, 73, 74]. Exposure to PM_2.5_ has also been linked to adverse respiratory symptoms and asthma during the cold season in the region [46, 73, 74].

The prevalence of asthma has been increasing worldwide and is a ubiquitous public health issue in the San Joaquin Valley. Two decades ago, 10.7% of the region’s population reported asthma symptoms, one of the highest rates reported in California [45], and the most recent California Health Interview Survey [68] shows that the prevalence of diagnosed asthma in the San Joaquin Valley is now 17.6% for all age groups, compared to 16% in the Bay Area and 15.1% in Los Angeles.

Furthermore, public health agencies have identified vulnerable populations, namely, ‘sensitive groups’, with chronic diseases and health conditions which include people with asthma, cardiovascular disease, and COPD, who are at increased risk of severe adverse outcomes from air pollution exposure [4, 10, 18, 20].

### Mitigation and control measures: adoption and adaptation

Few studies have investigated the application of community-, household-, and individual-level mitigation measures to protect health from the adverse effects of air pollution. Furthermore, there is scarcer evidence exploring the association between air pollution and health-related quality of life [33]. In terms of air pollution control and mitigation policies and interventions, there is a menu of economic instruments for air pollution control, such as emission trading permits and emission charges, and those instruments have been written about elsewhere [38], various approaches have been proposed to reduce the burden on health from poor air quality.

Recent episodes of high air pollution in SJV and climate change-related events that affect air quality [48, 59, 71] have compelled some public health agencies and healthcare providers to recommend policies and interventions that could reduce the risk of harm to human health. Nevertheless, it is not well understood whether people support them and whether individual-level factors mediate equitable adoption and adaptation.

Through the implementation of behavior change interventions and policies, public health agencies should aim to accomplish two goals: 1) protect people from being exposed, and 2) reduce overall exposure to outdoor air pollutants. The scoping evidence available in Table 1 describes application dimensions of air pollution protective policies and interventions that have been peer reviewed.

**Table 1:** Mitigation and exposure control measures and applications

Sofia et al. [62]	Carlsten et al. [11]	World Health Organization [72]	Allen & Barn [2]	James et al. [34]	Park et al. [52]
Reduce energy consumption for Individuals and Households	Fitted N95 masks	Emphasize risk communication for susceptible and vulnerable populations	Use HEPA filters for HVAC systems	Use HEPA filtration to improve asthma control	Use portable air purifiers
Use public and active transportation	Switch from motorized to active transportation	Stay indoors	Use portable air purifiers		
Change to efficient burning fuels	Choose routes to minimize NRAP and traveling during peak times	Avoid highly polluted places	Use well-fitted N95 masks		
Invest in internal climatization (Painting outer walls in light colors)	Drive with windows closed and maintain car air filtration	Reduce physical activity when health alerts are triggered	Stay indoors and Keep windows closed with AC		
Adopt diet to increase antioxidants intake	Avoid engine idling	Gather more evidence to measure consequence of exposure for high-risk groups	Balance indoor sources: cooking, heating, physical activity		
Improve efficiency of stock farming and manure management	Exercise regularly but moderate when pollution is high	Use of air filters in buildings and homes	Buildings with proper mechanical ventilation and filtration		
Electrify ports	Check air pollution levels	Use of respirators	Traveling through areas with less pollution		
Reduce fossil fuels for power generation	Use clean fuels at home and ventilate during cooking or heating		Walking and cycling		
Reduce work hours for congestion and travel time reductions	Use portable air cleaners		Use high efficiency cabin filters with windows closed		
Monitor pollutant levels and signal thresholds of safety	Effective clinical management of respiratory and cardiopulmonary diseases				
Adoption of electric or hybrid vehicles via tax incentives	Diet modification to include antioxidants and anti-inflammatory agents				

Additionally, some studies have gauged individuals’ disposition to accept environmental policies and behavior interventions around the world [1, 16, 50, 53, 55, 58], including assessing the influence of culture on minority groups and political affiliation effects on views and support to protect the environment.

### Aims and research questions

There is a gap in the scientific literature investigating favorability for these policies and interventions, and health-related quality of life costs particularly for populations and regions that are overburdened by poor air quality. The SJV differs substantially in topography, persistent air pollution concentration levels, and demographics across the countries in which similar studies have been conducted. Evidence is needed to understand support among residents in the SJV region and variation in socioeconomic and other factors. The central research questions investigated residents’ views of air pollution, explored favorability for policies and interventions that would mitigate air pollution harm, and identified factors that predict and explain adoption efforts to reduce air pollution and adverse health outcomes.

## Methods

### Data and participants

The online survey included responses from 310 adult participants and residents of zip codes in the San Joaquin Valley (SJV) region of California. The study included 34% of participants who had been diagnosed with asthma by a medical provider. The survey utilized the web-based software Qualtrics to distribute and collect responses through its online panel database, ensuring robust data management and security. Participants were adults aged 18 and older residing in zip codes located in the SJV and Qualtrics employed measures to prevent multiple submissions, including bot detection, cookies, and one-time-use survey links. The survey director monitored distribution in real-time until the minimum sample size of fully completed responses was reached. Qualtrics ensured the validity of the data by providing complete surveys with no missing data in the final dataset with no collection of sensitive or personal information from participants. The survey was available in English only; participants were compensated for their time at a rate of less than ten USD and were eligible to answer once. The study was approved by the IRB protocol #UCM2020-88 and registered under agreement with the EuroQOL research foundation.

### Study design

For the development of the survey instrument, extensive peer-reviewed literature [9, 14, 16, 50, 55, 70] was examined to build a comprehensive structure of air pollution mitigation and control measures and household- and individual-level behaviors for the region’s survey participants. The final survey included the following policy-relevant areas to assess in this study:

1. Personal, family, and home protections
2. Perceived health effects of air pollution
3. Point source emission mitigation and control: transportation, manufacturing, food and agriculture, and forest fires and wildfires.
4. Information availability, access, and awareness

The assessment asked about health status, self-protective behaviors, attitudes toward air pollution mitigation recommendations, air quality information knowledge, and mitigation behaviors, as well as whether their views were favorable or unfavorable.

The dependent variables in our models were health state (having asthma, one or more chronic diseases identified in the air quality sensitive groups, and not having asthma or any chronic diseases), political views (left- or right-wing ideologies), and support (level of agreement with air pollution policy and interventions). The independent variables included in the study were derived from existing environmental policy attitudinal literature and were gender, age, ethnicity, language spoken at home, income, residence distance to a freeway, and rurality.

### Quality of life distributional equity

The survey instrument included the quality-of-life assessment tool EuroQol-5D-3L. By investigating self-reported health outcomes, this instrument is essential for valuing health and making comparisons by health state to evaluate health-related quality of life (HRQoL).

The EuroQol-5D-3L (EQ-5D-3L) is a standardized instrument widely used to measure HRQoL by assessing five dimensions of health: mobility, self-care, usual activities, pain/discomfort, and anxiety/depression, each with three levels of severity. Utility values are derived by quantifying the preferences for different health states using the time trade-off (TTO) method. In TTO, participants are asked to balance the trade-off between living a longer duration in a less desirable health state versus a shorter time in perfect health, providing a utility score between 0 (equivalent to death) and 1 (perfect health). These utility values enable comparison of different health states and are instrumental in economic evaluations to inform healthcare and public health decision-making.

### Statistical analysis

Demographic characteristics are reported using descriptive analysis. To test the hypothesis of whether there are differences by health status, the likelihood ratio chi-square test of independence was conducted, and the *p* values were reported. Utility values were estimated using the results of the EQ-5D-3L survey [21], which is widely used to value health in health economics. To understand what factors, predict favorability of air pollution mitigation and control policies in the SJV region, the analysis included a stepwise regression with all the factors in the survey. All the unobserved factors reported as predictors were accepted at a *p* value ≤ 0.10 via the forward selection method.

Multivariate logistic regression was used to assess the characteristics of residents associated with health utility values, conditional on other covariates. Iterations were conducted for each latent factor to estimate the effects of the categorical variables sex, race, rurality, political views, marital status, proximity to the freeway, asthma, chronic conditions, having a child with asthma, being insured by a public health program, low-income level, and employment. Age, years of education, health utility values, and political views were included as continuous variables.

Finally, to explore what policies and interventions SJV residents support, principal component analysis (PCA) was used using varimax rotation. PCA is a type of multivariate analysis for feature extraction and observation of trends, clusters, and outliers. Each component is orthogonal by design and is appropriate for complex data while an extemporary theoretical framework applies in exploratory research, sampling adequacy, sphericity, and significance are reported by the Kaiser–Meyer–Olkin (KMO) and Bartlett’s test statistic. Predictors of latent factors were obtained using regression modeling to understand the characteristics, relationships, and magnitude of support for policies and interventions to mitigate air pollution exposure.

All the statistical analyses were performed using Stata v.17.

## Results

### Descriptive

The survey participants were adults 18 to 83 years of age who reside within zip codes in the San Joaquin Valley (SJV). The mean age of the whole sample was 42.90 (± 16.84) and 40.03 (± 14.92) years for people who have asthma themselves or have children with asthma, respectively. Table 2 summarizes the characteristics of the participants and reports the p-value for each reported characteristic of residents with and without asthma.

**Table 2:** Demographic characteristics of survey participants by asthma status

	Resident/Guardian without reported asthma		Resident with asthma or Parent/Guardian of Child with asthma		*p*-value
	n	%	n	%	
**Age**					
18–29	40	27.78	51	30.72	*p* = *0.000*
30–49	35	24.31	71	42.77	
50–64	34	23.61	33	19.88	
65 +	35	24.31	11	6.63	
**Sex**					
Female	92	63.89	109	65.66	*p* = *0.946*
Male	51	35.42	56	33.73	
Non-binary	1	0.69	1	0.60	
**Race/Ethnicity**					
Hispanic/Latino/Latina	43	32.09	53	32.32	*p* = *0.684*
White	68	50.75	86	52.44	
African American	5	3.73	7	4.27	
Asian/Pacific Islander	12	8.96	8	4.88	
Native American	6	4.48	10	6.10	
**Employment status**					*p* = *0.045*
Full Time	38	26.39	55	33.13	
Part Time	24	16.67	34	20.48	
Unemployed Looking	27	18.75	33	19.88	
Unemployed Not Looking	14	9.72	15	9.04	
Retired	32	22.22	15	9.04	
Disabled	9	6.25	14	8.43	
**Rurality**					*p* = *0.733*
Urban	84	58.33	100	60.24	
Rural	60	41.67	66	39.76	
**# Of chronic diseases**					*p* = *0.000*
No Conditions	59	40.97	8	4.82	
1	81	56.25	87	52.41	
2	3	2.08	60	36.14	
3	1	0.69	9	5.42	
4 or More	0	0.00	2	1.20	
**Health insurance**					*p* = *0.000*
Medi-Cal	17	11.87	42	25.28	
Medicare	39	27.08	16	9.63	
Medicare/Medi-Cal	20	13.89	45	27.11	
Private/Employment-based	49	34.03	56	33.73	
Uninsured	19	13.15	7	4.21	
**Tobacco user**	28	19.44	47	28.31	*p* = *0.069*
**Vape/E-cigarrette user**	10	6.94	28	16.87	*p* = *0.007*
**Political affiliation**					*p* = *0.272*
Democratic	57	39.58	74	44.58	
Republican	43	29.86	51	30.72	
Libertarian	3	2.08	2	1.20	
Green	0	0.00	4	2.41	
Other Party	4	2.78	4	2.41	
Don’t know	12	8.33	6	3.61	
I don’t support any party	25	17.36	25	15.06	

Among survey participants, the number of chronic diseases, health insurance type, employment status, and vaping use were significantly different between the entire sample population and people who were diagnosed with asthma or had a child who had asthma. Most respondents were female, White, spoke English and had the same distribution in the group of residents who had asthma. Hispanic/Latinos/Latinas accounted for approximately 32% of the entire sample and included residents with asthma. Most respondents in the whole sample reported being single (44.11%), but 54.26% of people with asthma reported being married or living with a partner and being employed full- or part-time. The entire sample included a higher proportion of retirees (22.22%) compared to the group with asthma (9.04%); this difference may be explained by the younger average age of individuals who reported an asthma diagnosis. Fifty-eight percent to 60% of the participants lived in an urban area, whereas 41.67% of the sample and 39.76% of the participants with asthma reported living in rural areas of SJV.

In the entire sample, 56.25% of residents reported having one chronic condition that is worsen by air pollution, and 4.82% of participants with asthma reported having only that diagnosed condition; the majority reported having one (52.41%) other diagnosed condition, and 36.14% had two other diagnosed chronic diseases. Most survey participants in both groups reported being insured by employer-based or private insurance, and 4.52% of people with asthma reported being uninsured. Notably, people with asthma reported tobacco use (28.31%), vape use (16.87%), and cannabis use (21.08%). The supplemental material contains the categories not included in Table 2, including income level that were analyzed for group differences.

Finally, political affiliation was not statistically significant (*p* = *0.272*) in measuring differences according to asthma status. SJV residents with asthma reported higher Democratic party affiliation (44.58%) compared to residents without asthma (39.58%). Similarly, Republican party affiliation was reported by people with asthma (30.72%), which was slightly higher than people without asthma in the sample (29.86%). The next highest reported political affiliation was no-party support among people without asthma (17.36%), which is slightly higher to people who had been diagnosed with asthma (15.06%).

### Air quality information symmetry

Chronic diseases were found to be significantly related to the likelihood of variability between groups of survey respondents. Thus, Table 3 (and supplemental material) describes the extent to which people with reported chronic diseases (CDs) perceive air pollution, assess air quality information in the region, and trust such information compared with people without reported chronic diseases (NCDs).

**Table 3:** Information symmetry by Health Status Group

	All	No Chronic Diseases	Chronic Diseases	*p*-value
	***N* = 310**	***n* = 205**	***n* = 105**	
	%	%	%	
**How informed do they feel about AQ information in your city?**				*p* = *0.02*
Not Informed at all	2.65	2.50	3.00	
Not very informed	15.00	11.00	23.53	
Somewhat informed	56.29	60.00	49.00	
Very informed	26.16	27.00	24.51	
Don’t know	-	-	-	
**Trust in Air Quality Information Sources**				*p* = *0.4*
Research institutes	5.6	3.37	9.10	
Universities	5.6	6.74	3.64	
Local Health authorities	25.00	26.97	21.82	
CBOs/Citizen groups	4.90	4.49	5.45	
Environmental protection agencies	13.89	13.48	14.55	
City government and municipalities	2.78	1.12	5.45	
Television or radio	28.47	31.46	23.64	
Internet	13.89	12.36	16.36	
**Time of day notice air pollution**				*p* = *0.01*
Morning	24.52	23.90	27.71	
Afternoon	40.65	40.49	40.95	
Evening	22.90	23.41	21.90	
All day is bad	18.39	20.98	13.33	
Don’t notice bad air	14.52	10.73	21.90	
**Season of the year notice air pollution**				*p* = *0.9*
Spring	25.81	24.88	27.62	
Summer	81.29	80.00	83.81	
Fall	29.03	31.71	23.81	
Winter	12.58	12.68	12.38	
**Method to check Air quality in their area**				*p* = *0.4*
Mountain range is clear	42.58	40.49	46.67	
Valley floor is clear	17.10	18.54	14.29	
Rely in recommendations from Air District	31.29	30.24	33.33	
AQ flags in my kids’ school	5.16	5.85	3.81	
Monitor at home	3.55	4.88	0.95	
Purple air website	10.32	12.68	5.51	*p* = *0.05*
Check the internet	14.19	13.17	16.19	
CalFire text messages	7.74	9.27	4.76	
I don’t check the Air Quality	10.65	8.78	14.29	
TV, Radio, or Newspaper	2.54	2.25	1.61	

Access to information, trust, and awareness were found to be statistically significant. Compared to residents without chronic diseases (NCDs), those with chronic diseases (CDs) who are sensitive to air pollution reported feeling the least informed about the quality of the air in their cities. Television, radio, and local health authorities were reported to be the most trusted sources of information by all groups, and more than half of all group respondents reported the information to be accurate and complete; 40% of people with chronic diseases (CDs) replied that the air quality information they received was accurate but incomplete.

In terms of air pollution awareness, all groups reported that most air pollution is perceived in the afternoon and in the summer season in SJV; this coincides with higher levels of ozone concentrations that are perceived by haze or smog during the warm season, which is endemic to the region. Most participants in each group responded that the method they use to check the air quality in their area is to observe if the mountain range in the region is clear (All: 42.58%, NCD: 40.49%, CD: 46.67%), with the highest proportion of people with chronic diseases indicating this as their primary option, followed by recommendations from the SJV Air District and checking the internet. Fourteen percent of people with chronic diseases reported not checking the air quality, whereas 8.78% of those with no chronic diseases did.

In contrast to the findings related to the time of day and season of the year for heightened awareness of air pollution, a high count of particulate matter (PM) in the air (All: 43.87%, NCD: 44.39%, CD: 42.86%), as well as mold/pollen presence (All: 39.35%, NCD: 38.54%, CD: 40.95%) were deemed to be the most likely meaning of a “bad air” day by all the groups alongside seeing the air dirty or polluted.

### Value of health

**Fig. 1:**
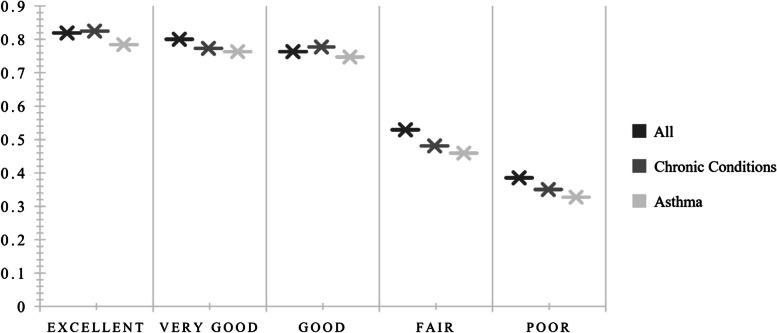
General health status by health utility state

All groups reported higher utility values when they responded that their current general health was excellent, and people with chronic conditions had the highest mean utility (*U* = 0.825). People with chronic conditions sensitive to air pollution also reported greater utility when their general health was good (*U* = *0.7*77) than did people with asthma (*U* = 0.747) or all (*U* = 0.763). People with asthma consistently reported the lowest mean utility values for all categories of general health status (see Fig. 1).

Table 4 reports the estimated mean utility values of each health state and the entire sample by age group, sex, ethnicity, education, number of chronic conditions, rurality, proximity to freeways/highways, and tobacco, vape, and cannabis use. The mean utility value for all participants was* U* = 0.710, whereas that for people with chronic conditions was *U* = 0.691, and the lowest value was reported by people with asthma (*U* = 0.657).

**Table 4:** Health utility values by condition

	All		Chronic Disease		Asthma	
	Mean	SD	Mean	SD	Mean	SD
Total	.710	.25	.691	.26	.657	.26
**Health Status**						
Excellent	.819	.21	.825	.20	.784	.24
Very good	.800	.19	.773	.21	.763	.22
Good	.763	.18	.777	.18	.747	.18
Fair	.529	.26	.481	.24	.459	.21
Poor	.385	.32	.350	.29	.327	.33
**Age**						
18–25	.707	.22	.707	.23	.711	.20
26–39	.693	.26	.667	.27	.646	.28
40–59	.683	.26	.662	.27	.625	.28
60 +	.778	.21	.755	.22	.683	.25
**Sex**						
Male	.740	.25	.734	.26	.692	.27
Female	.696	.24	.670	.25	.641	.25
Nonbinary	.573	.14	.573	.14	.471	-
**Ethnicity**						
Hispanic	.756	.21	.730	.22	.706	.21
Non-Hispanic	.690	.26	.673	.27	.634	.28
**Education**						
Low	.560	.34	.499	.44	.527	.42
Mid	.686	.25	.663	.25	.621	.25
High	.777	.22	.759	.22	.737	.24
**Number of conditions**						
None	.771	.23	-	-	.683	.23
1	.721	.23	.726	.24	.681	.25
2	.650	.26	.614	.28	.647	.27
3	.617	.27	.617	.27	.578	.25
4 +	.135	.28	.334	-	.135	.28
**Area**						
Urban	.704	.25	.760	.20	.746	.21
Rural	.719	.24	.767	.22	.735	.23
**Lives within 1-mile of Freeway**						
Yes	.696	.26	.667	.27	.636	.27
No	.736	.22	.729	.22	.695	.23
**Tobacco use**						
Yes	.664	.26	.666	.25	.628	.28
No	.725	.24	.700	.26	.668	.25
**Vape Use**						
Yes	.609	.24	.584	.27	.594	.23
No	.725	.24	.708	.25	.670	.26
**Cannabis use**						
Yes	.645	.28	.621	.29	.585	.29
No	.726	.24	.707	.25	.676	.25

The trend of general health status was followed in the estimated mean utility values by age group. People 60 years of age and older in the entire sample obtained the highest utility value for all (*U* = 0.778), and people 60 + years of age had the highest utility value for those with chronic diseases (*U* = 0.755). However, for people with asthma, the younger group (aged 18–25 years) reported the highest mean utility (*U* = 0.711).

Males reported higher utility values than females did and nonbinary residents of the SJV in all health states. People who reported high levels of education (a bachelor’s degree and above) also reported the highest utility for their current health. In terms of the number of chronic conditions, people with no chronic conditions reported the highest utility value (*U* = 0.771) compared with people with asthma, who reported comorbidities that were worsened by air pollution (*U* = 0.135).

People who live in rural areas in SJV and who have chronic conditions had greater health utility (*U* = 0.767), but people with asthma living in urban areas reported greater utility (*U* = 0.746) than did those with asthma living in rural areas of the Valley. Those residents who reported not living within one mile of a freeway or highway reported higher utilities in every health state, as did those residents who were not tobacco, vape, or cannabis users.

**Fig. 2:**
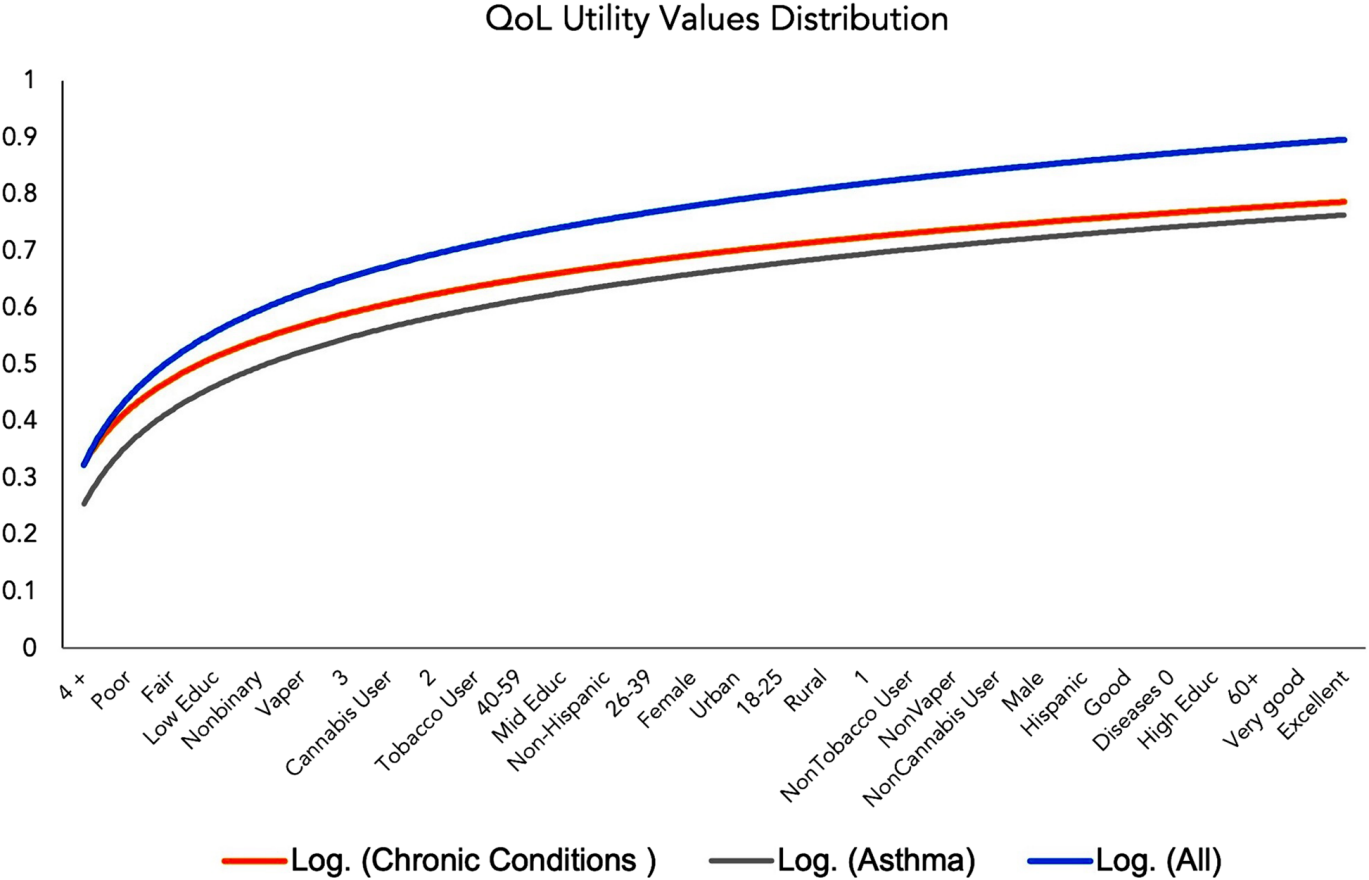
Health utility curves by health state

Using the logarithmic scale transformation for estimated utilities, Fig. 2 shows the utility curves for each health state to compare the health outcomes of all residents, people with chronic conditions, and people with asthma. In this analysis, the utility curve for people with asthma was lower than the curve for people with chronic conditions sensitive to air pollution and all the residents of the San Joaquin Valley.

### Favorability of policies and interventions

To determine which air pollution mitigation interventions are favored by SJV residents, an exploratory factor analysis was conducted using maximum likelihood estimation with fifty-two (52) policy items sorted into five thematic matrices. Primary components were rotated using varimax rotation to allow for factors to correlate. Tables 5, 6, 7, 8, and 9 show the factor loadings for each matrix after rotation, and acceptability means with corresponding standard deviations (SDs).

**Table 5:** Hybrid and electric car concerns rotated factor loadings (Varimax)

		Factor 1	Factor 2	Factor 3	Acceptability Mean (SD)
Too expensive		0.226	**0.656**	0.282	3.43 (1.11)
Too complicated to purchase		0.351	**0.697**	0.358	2.61 (1.43)
Charging stations are not widely available in my city		**0.727**	0.397	0.328	3.28 (1.17)
Charging stations are not widely available when I travel long distances		**0.934**	0.239	0.266	3.41 (1.11)
Too small for my family/Not spacious enough		0.251	0.303	**0.651**	2.59 (1.39)
Not safe		0.200	0.322	**0.763**	2.60 (1.49)
Don’t like the look		0.186	0.291	**0.816**	2.32 (1.38)
Don’t go fast enough		0.244	0.170	**0.854**	2.27 (1.43)
KMO = 0.856					
Bartlett’s test	Approx. Chi-square = 1533.2				
	df = 36				
	Sig. = 0.000

**Table 6:** Effectiveness of organizations in improving air quality rotated factor loadings (Varimax)

		Factor 1	Factor 2	Acceptability Mean (SD)
U.S. Government		**0.638**	0.227	2.21 (0.99)
EPA		**0.876**	0.003	2.85 (0.96)
State of California		**0.800**	0.194	2.68 (0.99)
SJV Air Pollution Control District		**0.612**	0.567	2.84 (0.94)
County/City Officials		0.520	**0.609**	2.26 (0.98)
Farmers, Growers, Ranchers		0.053	**0.876**	2.65 (1.10)
Community Grassroots organizations		0.269	**0.822**	2.57 (0.97)
Environmental organizations		**0.730**	0.357	2.97 (0.96)
Scientists and Researchers		**0.675**	0.373	3.09 (0.90)
KMO = 0.784				
Bartlett’s test	Approx. Chi-square = 1252.0			
	df = 36			
	Sig. = 0.000

**Table 7:** Cost of policies and factors to consider rotated factor loadings (Varimax)

		Factor 1	Factor 2	Acceptability Mean (SD)
Costs that it will impose on businesses		**0.810**	-0.129	3.06 (0.91)
Cost to consumers		**0.847**	-0.105	3.36 (0.99)
Reducing PM_2.5_		0.458	**0.523**	3.23 (0.99)
Reducing ozone		0.422	**0.534**	3.42 (1.07)
Making the air look cleaner		**0.538**	0.420	3.37 (1.11)
Having it be locally run as opposed to by the EPA		**0.653**	0.219	2.91 (1.06)
The impact it will have on low-income communities		**0.666**	0.276	3.45 (1.06)
Making sure that people in the Bay Area also have to reduce their air pollution		**0.578**	0.331	3.66 (1.01)
Getting agriculture to change its practices		0.084	**0.778**	3.15 (1.04)
Reduce heating from fireplaces or woodstoves		0.121	**0.730**	2.95 (1.20)
Eliminate or reduce large incinerators		0.155	**0.770**	3.25 (1.13)
Reduce the amount of pollution from commercial cooking		0.048	**0.814**	2.89 (1.09)
Reducing pollution from dairies		0.035	**0.841**	3.09 (1.20)
Eliminate pesticide drift		0.262	**0.685**	3.68 (0.97)
KMO = 0.818				
Bartlett’s test	Approx. Chi-square = 2282.1			
	df = 91			
	Sig. = 0.000

**Table 8:** Mitigation and control policy statements rotated factor loadings (Varimax)

		Factor 1	Factor 2	Acceptability Mean (SD)
“During the COVID-19 period or shelter-in-place, I have sensed/experienced good clean air and I don't want to go back to the air pollution levels we previously had”		0.306	0.433	3.22 (1.27)
“Cities must take effective measures to protect citizens from air pollution, even if this requires reallocating public space to walking, cycling and public transport”		**0.767**	0.326	3.59 (1.21)
“Cities must take effective measures to protect citizens from air pollution, even if it means preventing polluting cars from entering the city”		**0.743**	0.256	3.23 (1.29)
“I think we need to resume our normal lives soon and must accept that air pollution will rise again”		-0.522	0.582	3.07 (1.32)
“More public space in your nearest town/ city should be reserved for public transport (e.g., by expanding bus lanes)”		0.316	**0.690**	3.30 (1.05)
“More public space in your city and nearest towns should be reserved for cycling”		0.415	**0.742**	3.51 (1.03)
“More public space should be reserved for pedestrians”		0.380	**0.724**	3.62 (1.00)
“More policies should be adopted to stop polluting cars and trucks from entering the city, for example through Zero-Emission Zones”		**0.743**	0.269	3.28 (1.21)
“More companies should allow their workers to continue working from home or telecommuting”		**0.542**	0.288	3.96 (0.98)
“More cities and counties should work together to create clean, fast and reliable regional transportation systems to connect the Central Valley”		**0.714**	0.250	4.11 (1.01)
KMO = 0.820				
Bartlett’s test	Approx. Chi-square = 1407.7			
	df = 45			
	Sig. = 0.000

**Table 9:** Persistence of poor air effects rotated factor loadings (Varimax)

		Factor 1	Factor 2	Acceptability Mean (SD)
Breathlessness/having more difficulty in breathing		**0.689**	0.330	2.73 (1.09)
Doing less outdoor activities		**0.667**	0.387	3.13 (1.27)
Doing more to look after my skin		0.361	**0.731**	2.87 (1.24)
Doing more to stay healthy		0.180	**0.793**	3.29 (1.17)
Feeling Depressed		**0.653**	0.214	2.69 (1.23)
Irritation to eyes/nose/throat		**0.736**	0.138	3.21 (1.16)
Skin problems		**0.741**	0.349	2.45 (1.23)
Wanting to move to other less polluted place		**0.706**	0.207	2.91 (1.44)
Asthma incidents or attacks		**0.747**	0.139	2.18 (1.28)
Poor visibility		**0.700**	0.126	2.57 (1.14)
Worrying about the environment for children and elderly		**0.588**	0.277	2.97 (1.39)
KMO = 0.891				
Bartlett’s test	Approx. Chi-square = 2922.4			
	df = 78			
	Sig. = 0.000			

Factor Loadings: Hybrid and Electric Car Concerns (Table 5).

- Factor 1 = Charge
- Factor 2 = Expense
- Factor 3 = Car attributes

The items loading into the first factor related to hybrid and electric care adoption suggest that this component corresponds to concerns about electric charge access and availability in SJV cities and when traveling long distances. The second factor represents the expense concerns that residents have in acquiring and adopting clean cars, in which these vehicles are too expensive, and the purchase process is perceived as onerous. The third factor is related to hybrid and electric vehicle attributes.

Factor Loadings: Effectiveness of Organizations to Improving Air Quality (Table 6).

- Factor 1 = Not Local
- Factor 2 = Local

In assessing the effectiveness of organizations working to improve air quality in SJV, as shown in Table 6, a two-factor solution was recommended for parallel analysis. The first factor corresponded to organizations that are largely perceived as participating from a non-local perspective but rather from national, state, and regional levels, which included the SJV Air Pollution Control District. The second factor correlated with stakeholders with local influence, such as city and county officials, farmers, growers and ranchers, and community-based organizations.

Factor Loadings: Cost of Policies and Factors to Consider (Table 7).

- Factor 1 = Cost
- Factor 2 = Who Pays

This study asked about factors and costs that need to be considered when formulating air pollution control and mitigation policies for SJV residents. The midpoint for this scale was 3, and most of the mean acceptability indicators strongly support the inclusion of these considerations in policymaking. Two solution-correlated factors were identified: the cost to businesses and consumers, as well as the goal of making the air visibly cleaner, to guide local policymaking. Other consideration was the impact on low-income communities and the need for the neighboring Bay Area to reduce its air pollution, which often travels and accumulates in the SJV basin. A second factor correlated the reduction in fine particulate matter (PM_2.5_) and ozone, with agricultural practices to reduce air pollution, reduce heating from fireplaces, eliminate, or reduce pollution from large incinerators, commercial cooking, dairies, and pesticide drift.

Factor Loadings: Mitigation and Control Policy Statements (Table 8).

- Factor 1 = Air Pollution Control
- Factor 2 = Public Space

The strongest support was reported in the section on policy mitigation and control statements. The scale also had a midpoint of 3, and all attitudes were favored with the highest mean acceptability for the creation of a clean, fast, and reliable regional transportation system to connect SJV residents and their communities. A component correlated statements directly addressing strong action for air pollution control to protect the health of SJV residents. The second factor corresponds to the preservation of public space for public transportation, cycling, and pedestrians.

Factor Loadings: Persistence of Poor Air Effects (Table 9).

- Factor 1 = Symptoms
- Factor 2 = Personal Actions

Finally, this study sought to understand the degree to which air pollution influences the persistence of disease outcomes and self-protective behaviors. Factor 1 correlated symptoms related to poor air, such as difficulty breathing; engaging in fewer outdoor activities; feeling depressed; irritation to one’s eyes, nose, and throat; skin problems; wanting to move to a less polluted place; asthma exacerbations; poor visibility; and worries about the environment that SJV children would inherit and are harming the elderly in the region. The second factor corresponds to having to do more to care for one’s skin and to stay healthy overall.

### Predictors of support and unfavorability

Age had a positive predictive relationship with all the latent factors reported in Table 10, except for experiencing more symptoms related to poor air quality. Increases in years of education favor charges, expense, and car attributes as concerns for the adoption of hybrid and electric vehicles, as well as experiencing more symptoms due to poor air and having to take more personal actions to protect themselves. Decreases in estimated health utility values predicted favorability for all latent factors with the highest magnitude for experiencing symptoms due to bad air. Higher utilities were positively associated with supporting agencies such as the EPA, the state, and the U.S. government to control and mitigate air pollution in the region, but the finding was not statistically significant.

**Table 10:** Predictors of latent factors

Predictors	Charge		Expense		Car attributes		Not Local		Local		Cost		Who pays		Air pollution control		Public space		Symptoms		Personal actions	
	β (SE)		β (SE)		β (SE)		β (SE)		β (SE)		β (SE)		β (SE)		β (SE)		β (SE)		β (SE)		β (SE)	
Age (c)	.009*	(.00)	.016**	(.00)	.003	(.00)	.000	(.00)	.002	(.00)	.007*	(.00)	.002	(.00)	.001	(.00)	.001	(.00)	-.003	(.00)	.004	(.00)
Male	-.448**	(.16)	-.303*	(.14)	-.323*	(.15)	.172*	(.08)	.133	(.10)	-.045	(.08)	-.092	(.08)	.005	(.10)	.085	(.10)	-.270**	(.08)	-.358**	(.12)
NH White	-.053	(.13)	-.282*	(.14)	-.165	(.15)	-.163	(.09)	-.174	(.10)	-.076	(.09)	-.115	(.09)	-.109	(.10)	-.156	(.15)	.100	(.08)	.008	(.12)
Rural	-.156	(.13)	.003	(.13)	.258*	(.14)	.004	(.08)	-.024	(.10)	.044	(.08)	-.072	(.08)	-.006	(.09)	.073	(.10)	-.062	(.14)	.027	(.12)
Political Views (c)	-.071*	(.03)	-.019	(.03)	-.124**	(.04)	.040	(.02)	-.059*	(.03)	-.008	(.02)	.155***	(.02)	.161***	(.03)	.139***	(.03)	.068**	(.02)	.111**	(.03)
Married	-.009	(.13)	-.134	(.15)	-.106	(.14)	.110	(.09)	.174	(.10)	.110	(.09)	.204*	(.09)	.011	(.10)	.117	(.10)	.143	(.08)	.116	(.12)
Lives 1-mile from Freeway	-.107	(.13)	-.138	(.13)	-.071	(.14)	.141	(.08)	.203*	(.10)	.031	(.08)	.154*	(.08)	-.048	(.09)	-.135	(.10)	.143	(.08)	.110	(.12)
Years of Education (c)	.034	(.13)	.208	(.14)	.237*	(.14)	-.013	(.08)	-.138	(.10)	-.007	(.08)	-.105	(.08)	-.067	(.10)	-.112	(.10)	.162*	(.08)	.103	(.12)
Health Utility Value (c)	-.163	(.28)	-.274	(.30)	-.031	(.30)	.155	(.18)	-.075	(.20)	-.051	(.18)	-.034	(.18)	-.079	(.21)	-.046	(.21)	-1.06***	(.17)	-.098	(.25)
Have Asthma	-.025	(.16)	-.050	(.16)	.087	(.17)	.118	(.10)	.068	(.11)	.206*	(.10)	.143	(.10)	.016	(.11)	.167	(.12)	.447***	(.09)	.336*	(.14)
Have Chronic Diseases	-.032	(.09)	-.126	(.10)	-.186*	(.10)	-.027	(.06)	-.085	(.07)	-.034	(.06)	.045	(.06)	.117	(.07)	-.090	(.07)	.036	(.05)	.024	(.08)
Child has Asthma	-.031	(.08)	.028	(.08)	.147*	(.08)	.010	(.05)	-.019	(.06)	.030	(.05)	.001	(.05)	-.025	(.06)	.093	(.06)	.182***	(.05)	.277***	(.07)
Public Health Insurance	-.132	(.14)	-.141	(.15)	.131	(.15)	.083	(.09)	.260*	(.11)	-.073	(.09)	.035	(.09)	-.012	(.11)	-.170	(.11)	.015	(.08)	-.047	(.13)
Low Income	-.073	(.16)	.023	(.08)	.135	(.18)	-.035	(.10)	.462***	(.08)	.129	(.11)	.016	(.10)	-.041	(.12)	.089	(.12)	-.032	(.10)	.125	(.14)
Employed	-.134	(.14)	.071	(.14)	-.005	(.15)	-.106	(.14)	.228*	(.11)	-.045	(.09)	-.115	(.09)	-.016	(.10)	-.050	(.11)	.044	(.09)	-.006	(.13)

Levels of significance:

^*^p < .05; **p < .01; ***p < .001

Five latent factors were negatively predicted by political views, indicating that, compared with liberal residents, neutral to more conservative leaning SJV residents were more likely to prefer non-local organizations to regulate air quality, to favor polluters to pay for their contamination, for strong air pollution control regulation, preservation of public space, and to report more symptoms or having to take more personal protective actions due to poor air quality. However, being low income, being fully employed, and insured by a public health program predicted favorability to having local agencies, such as farmers and ranchers, community-based organizations, and city/county governments regulate and control air pollution in the region. Living 1-mile from a freeway or highway followed those same predictions and favored who pays for the pollution that is produced in the region.

Males, non-Hispanic whites, and people who are employed negatively predict the acceptability of most factors except for males and people who are employed because of the effectiveness of local and non-local organizations; air pollution control measures; and the preservation of public space for pedestrians, cyclists, and public transportation. Whites were likely to have pervasive symptoms related to poor air and to take more personal protective actions to safeguard their health.

Having a child with asthma and rurality positively predicted the importance of car attributes in the adoption of hybrid and electric vehicles. Having asthma and having a child with asthma were also significant factors in predicting experiences of air quality-related symptoms and having to do more to protect their health. Nevertheless, having asthma is favorable for accounting the costs that air pollution control will impose on businesses and consumers.

## Discussion

This research aimed to evaluate the distributional equity of health-related quality of life, understand how San Joaquin Valley (SJV) residents assess air quality, what air pollution mitigation efforts and interventions are favored in the region, and which factors predict differential responses in their support.

This study results indicate that the residents of the SJV region report a sensory relationship with the state of air quality in their community, as most participants reported observing the clarity of the mountain range and valley floor as their preferred way to check air quality. This is consistent with findings in studies of environmental risk [22, 61, 69], where sensory evaluation has been associated with perceptions of climate change, personal health, and public health adverse effects from exposure, such as noise pollution. The interaction of sensorial identification of exposure risks could be a factor that explains why most SJV residents in this study perceive summer as the season of the year and the afternoon as the time of day with the highest pollution. The presence of nitrogen dioxide and ground-level ozone, with their conspicuous hazy characteristics in the warm season, mediates this relationship. This finding may also be responsible for moderating the response to mitigate exposure during the region’s winter season, in which “invisible” PM_2.5_ levels are pervasive; this is critically important since regional studies have demonstrated that even short-term exposure to dangerous PM_2.5_ is associated with emergency department visits and hospitalizations for asthma and respiratory infections [65, 73, 74].

Furthermore, this evidence demonstrates the existence of robust regional identity in the concern of air pollution among SJV residents. In scientific examinations of general environmental policy support, researchers have investigated the relationship between environmental concerns and the favorability of policies, and the findings support that regional identity is a driving factor for pro-environmental attitudes and, to a degree, could lead to potential behavior change [8, 12, 40, 49]. Seemingly, a potential avenue facilitating this common stance is the dissemination of air quality information through radio and news, in which the relational attribution of air pollution concentrations in one community and its application to others within the region may occur.

Of particular importance are differences found between people with chronic diseases and those without. Residents who had one or more chronic conditions sensitive to air pollution were found to report differences in how informed they feel about the air quality of their community, the time of the day when they notice air pollution, access to internet tools to obtain real-time air pollution data (Purple Air) and knowing what a “bad air” day means. These differences are consistent with a previous study [70] that recommended the need to increase outreach to vulnerable populations to whom air pollution is causing increased susceptibility to adverse health outcomes. Removing limitations of awareness with accurate and complete availability of air pollution data by community-defined areas would better serve the mediation between the “localization” of air quality information and residents’ risk assessment [7].

In analyzing whether air pollution elicits differential responses from groups in the SJV region, people who reported having asthma or having a child with asthma presented a log-scale health utility curve that was lower than that of people with chronic conditions and the entire sample. The study findings demonstrate that air pollution-associated health outcomes, such as asthma, are associated with higher health-related quality of life (HRQoL) costs than other chronic conditions are.

To the best of our knowledge, this evidence is the first of its kind for the SJV region to incorporate utility-based quality of life assessment to measure the impact of air pollution on populations in different health states. This study adds to the body of knowledge investigating HRQoL for people with asthma or chronic conditions, as well as their perceptions and policy support [63, 64].

### Policy favorability

On the basis of a wide range of mitigation and control air pollution policies, the results indicate that the current policy and intervention environment in the SJV can be explained by eleven (11) factors or concerns in the domain of air pollution control and mitigation: 1) hybrid and electric vehicle (HEV) charge, 2) HEV expense, 3) HEV attributes, 4) local organizations regulating emissions, 5) non-local organizations regulating emissions, 6) the costs of reducing air pollution in the region, 7) who pays for those costs, 8) air pollution control, 9) preservation and promotion of public space, 10) health symptoms from air pollution exposure, and 11) personal actions taken to protect one’s own health from air pollution damage.

Whereas the majority of existing research suggests that ideology and socioeconomic factors predict the acceptability of environmental policies [23, 30], this analysis indicates that the health state of SJV residents is also a significant predictor of favorability and concern for air pollution control and mitigation policies and interventions. Thus, people who have asthma and those who have children with asthma seem to be highly concerned with the adverse health effects and symptoms that air pollution produces, as well as the need to do more to maintain their health while living in these environmental conditions. Moreover, health-related quality of life (HRQoL), as measured by health utility, appears to be a negative predictor for almost all air pollution concern categories and a positive predictor in favor of regulating air pollution emissions by local organizations.

In the existing body of knowledge, political ideology has been found to be deterministic in the acceptability or rejection of environmental policy. Whereas right-wing or conservative leanings are suggested to reject policies to protect the environment [16, 29], this evidence suggests that right-wing ideology is a negative predictor of HEV adoption concern due to charge infrastructure availability, HEV attributes, and having local organizations intervene in air pollution emissions. Left-wing ideologies predict favorability for policies that charge polluters to clean the air, control air pollution emissions, preserve public space, and implement interventions that reduce air pollution health symptoms and ease the burden on health.

### Gaps and limitations

California’s San Joaquin Valley (SJV) is a region where Latinos/Latinas/Latinx constitute 50.2% of the approximately 4.3 million residents, and our sample did not reflect this racial and ethnic composition, and given the vulnerability of specific groups (i.e., disadvantaged and underserved neighborhoods in the state identified as facing the worst air pollution and environmental health burdens, such as AB617 in California) and the heterogeneity of communities in the region, future research should increase the participation of people of color and underrepresented communities in the estimations of air pollution health-related costs in quality of life and policy favorability. The limited sample size, constrained by the lack of funding, restricts the ability to fully capture the diversity of the region's population. Future research with broader and more inclusive sampling methods is needed to validate these findings and enhance representativeness. Furthermore, the measurements presented in the context of evaluating environmental policy related to air pollution need to be replicated in regional studies to examine their validity in representing orthogonal constructs involved in air pollution.

### Recommendations for future research

While this study is a leap forward in establishing an empirical organization of various air pollution policies and interventions, there is still a long way in investigating the potential drivers, acceptance, and sustainability in a range of contexts within the SJV region that would facilitate improvement of this endemic public health issue. As with any policy and intervention, evaluation in the context of real-life dimensions for SJV is vital for ensuring adherence and sustaining behavior change. Researchers generally accept that cost-effectiveness evidence to estimate the value of routinely suggested individual, household, and behavior change strategies is highly needed and currently scarce. As health economics makes inroads in public health decision-making, it is imperative to study the cost-effectiveness of pollution mitigation interventions and policies.

In reviewing the available literature [13, 24, 25, 27, 32, 35, 36, 39, 41, 42, 44, 47], most cost-effectiveness studies emphasize the need for the inclusion of diverse communities, short- and long-term support, and availability of the suggested pollutant exposure reduction approaches.

Carefully designed studies are needed to determine how different mitigation strategies and interventions could play a role in complementing existing regulations to continue reducing air pollution emissions and increasing the efficiency of public health protection in the short and mid-term while considering compounding crises.

### Implications for policy makers

Efforts to regulate air quality have been evaluated and found to have an economic value of $2 trillion since inception until 2020 in terms of air quality improvements, indicating that its benefits outweigh its costs [43]. The Office of Management and Budget estimated in 2018 that the range of costs in the Obama presidential era for environmental protection policies was between $88 and $598 billion, whereas the benefits were between $219 and $695 billion [51]. Although these policies have not lacked substantial criticism, measures such as the catalytic converter in the 1960s and pivotal updates to the Act in the 1990s [66] have been deliberated and implemented to address environmental concerns such as acid rain, toxic air emissions, stratospheric ozone depletion, and the effects of urban air pollution and haze.

The San Joaquin Valley Air Pollution Control District is the public health entity designated to enforce structural standards and develop local plans to implement air pollution control measures. The air district is governed by an appointed board made of elected officials and two public members appointed by the governor [56]. On the other hand, local county public health departments are also commissioned to protect the public’s health, prevent illnesses, monitor and surveil, and respond to endemic factors that complicate the attainment of their constituency’s full health. Several local public health departments in Fresno, Madera, and Tulare Counties reported that their communities would like air pollution and asthma to be addressed while gathering data and providing feedback as part of National Public Health Accreditation efforts. This health inequity should be prioritized by the public health system in the region.

Furthermore, air pollution, as the main driver of climate change, has imposed new challenges in decreasing the burden of disease in regions where novel and long-standing environmental injustices have created inequities in the distribution of both bad air and equitable access to mitigation measures.

## Conclusion

This study provides analytical evidence of distributional costs related to endemic polluted air in the San Joaquin Valley (SJV) region of California. People with asthma and chronic conditions report higher costs in terms of quality of life. Local policymakers could invest in cohesive and democratic public health strategies that encourage trust in agencies and stakeholders, with the end goal of promoting equitable adoption of individual, household, and community protective policies and behavior change. Efficacious decisions are to be made under the air quality symmetry of information, but this evidence indicates that asymmetric information conditions exist and influence the state of public health.

Ostensibly, climate change is poised to increase the relative impact of outdoor air pollution on human health, and although there is no substitute for systemic environmental protections to control the sources and quantity of emissions, these policies tend to attain benefits in the long term. Public health practitioners and researchers have much more to do in the expedient implementation and evaluation of approaches to protect health in the short and medium term. Furthermore, these are timely observations, particularly for areas of the country enduring ubiquitous bad air and where lately, additional events of high pollution, such as forest fires and wildfires, have occurred.

Whether at the macroeconomic (structural) or microeconomic (individual and household) level, health economics offers a lens through which the preferences of decision-makers, health outcomes, and health costs can be assessed to adopt cost-effective mitigation and control measures of air pollution and avoid adverse health outcomes.

## Supplementary Information

Supplementary Material 1.

## Data Availability

Availability of Data and Materials: Data are available on request owing to privacy and ethical restrictions. The data presented in this study are available upon request from the corresponding authors.
