# Supplementary material for: Assessing public support for air pollution mitigation and control policies: health, socioeconomic, and ideological predictors in an overburdened and vulnerable region of the U.S
Source: BMC Public Health. 2025 Jan 22;25:263. doi: 10.1186/s12889-025-21366-7 (PMC11752818; doi:10.1186/s12889-025-21366-7)
Supplement: Supplementary file 1 — Supplementary Material 1. [file 12889_2025_21366_MOESM1_ESM.docx]

Table 2. Demographic Characteristics of Survey Participants by Asthma Status

|  | Resident/Guardian without reported asthma | | Resident with asthma or Parent/Guardian of Child with asthma | |  |
| --- | --- | --- | --- | --- | --- |
|  | n | % | n | % | p value |
| **Household Income** |  |  |  |  |  |
| Low | 43 | 29.86 | 47 | 28.31 | *p=0.497* |
| Middle | 67 | 46.53 | 70 | 42.17 |  |
| High | 34 | 23.61 | 49 | 29.52 |  |
| **Language in Household** | |  |  |  | *p=0.805* |
| English | 132 | 91.67 | 150 | 90.36 |  |
| Spanish | 8 | 5.56 | 13 | 7.83 |  |
| Hmong | 1 | 0.69 | 1 | 0.60 |  |
| Native American/Indigenous | 3 | 2.08 | 2 | 1.20 |  |
| **Education** |  |  |  |  | *p=0.756* |
| High School or below | 5 | 3.47 | 6 | 3.61 |  |
| Some College/ 2-year degree | 96 | 66.67 | 104 | 62.65 |  |
| College degree or Graduate | 43 | 29.86 | 56 | 33.73 |  |
| **Marital Status** |  |  |  |  | *p=0.103* |
| Married/Domestic Partner | 58 | 38.39 | 89 | 54.26 |  |
| Single/Widowed | 61 | 44.11 | 50 | 30.18 |  |
| Separated/Divorced | 25 | 17.78 | 27 | 15.13 |  |
| **Lives 1-mile from Freeway/Highway** | |  |  |  | *p=0.721* |
| Yes | 90 | 62.50 | 107 | 64.46 |  |
| No | 54 | 37.50 | 59 | 35.54 |  |
| **Cannabis User** | 23 | 15.97 | 35 | 21.08 | *p=0.250* |

Table 3. Information Symmetry by Health Status Group

|  | | All | | No Chronic Diseases | Chronic Diseases | p-value |  |
| --- | --- | --- | --- | --- | --- | --- | --- |
|  | | **N=310** | | **n=205** | **n=105** |  |  |
|  | | % | | % | % |  |  |
| **Rate Air Quality Information received** | |  | |  |  | *p=0.9* |  |
| Inaccurate and Incomplete | | 3.69 | | 3.76 | 3.53 |  |  |
| Accurate but Incomplete | | 38.00 | | 37.10 | 40.00 |  |  |
| Accurate and Complete | | 58.30 | | 59.14 | 56.47 |  |  |
| Don’t know | | - | | - | - |  |  |
| **Air quality in their city for past 2-weeks** | |  | |  |  | *p=0.1* |  |
| Very unhealthy | | 6.47 | | 8.29 | 2.88 |  |  |
| Unhealthy | | 17.15 | | 17.56 | 16.35 |  |  |
| Unhealthy for sensitive groups | | 26.86 | | 28.29 | 24.04 |  |  |
| Moderately healthy | | 35.60 | | 31.71 | 43.27 |  |  |
| Good air quality | | 13.92 | | 14.15 | 13.46 |  |  |
| **Current Air quality Assessment** | |  | |  |  | *p=0.3* |  |
| Very unhealthy | | 13.87 | | 16.10 | 9.52 |  |  |
| Unhealthy | | 21.94 | | 23.41 | 19.05 |  |  |
| Unhealthy for sensitive groups | | 26.45 | | 24.88 | 29.52 |  |  |
| Moderately healthy | | 26.77 | | 24.39 | 31.43 |  |  |
| Good air quality | | 10.97 | | 11.22 | 10.48 |  |  |
| **Meaning of “Bad Air” day** | |  | |  | |  | *p=0.6* |
| High mold/pollen levels in the air | | 39.35 | | 38.54 | | 40.95 |  |
| High PM counts in the air | | 43.87 | | 44.39 | | 42.86 |  |
| High Ozone levels in the air | | 17.42 | | 18.05 | | 16.19 |  |
| The air is gray or brown haze outside | | 43.55 | | 43.41 | | 43.81 |  |
| The hole in the ozone layer has made it unhealthy to be outside | | 5.81 | | 6.83 | | 3.81 |  |
| The air is dirty/polluted | | 59.86 | | 60.00 | | 59.05 |  |
| I don’t know what “Bad Air” days mean | | 1.61 | | 0.49 | | 3.81 | *p=0.02* |
